# Based on the immune system: the role of the IL-2 family in pancreatic disease

**DOI:** 10.3389/fimmu.2025.1480496

**Published:** 2025-01-31

**Authors:** Yuqing Zhu, Zheng Lu, Zhuo Wang, Jiao Liu, Ke Ning

**Affiliations:** College of Exercise and Health, Shenyang Sport University, Shenyang, China

**Keywords:** pancreatitis, pancreatic cancer, IL-2 family, IL-2, immunity

## Abstract

The IL-2 family, consisting of IL-2, IL-4, IL-7, IL-9, IL-15 and IL-21, is a key regulator of the immune response. As an important endocrine and digestive organ, the function of the pancreas is regulated by the immune system. Studies have shown that each cytokine of the IL-2 family influences the occurrence and development of pancreatic diseases by participating in the regulation of the immune system. In this paper, we review the structural and functional characteristics of IL-2 family members, focus on their molecular mechanisms in pancreatic diseases including acute pancreatitis, chronic pancreatitis and pancreatic cancer, and highlight the importance of the related proteins in the regulation of immune response and disease progression, which will provide valuable insights for new biomarkers in pancreatic diseases, early diagnosis of the diseases, assessment of the disease severity, and development of new therapeutic regimens. The insights of the study are summarized in the following sections.

## 1 Introduction

The interleukin-2 (IL-2) family consists of six cytokines, including IL-2, IL-4, IL-7, IL-9, IL-15, and IL-21, which share a γ-chain structural domain. These molecules are indispensable regulators in both innate and adaptive immune responses (1). IL-2 has multiple functions that promote T cell proliferation, enhance the cytotoxicity of natural killing (NK) cells, and promote the production of inflammatory factors by activating transcription factors such as STAT5 (2). In addition, other members of the IL-2 family have been shown to play key regulatory roles in the immune system (3).

Pancreatitis is an inflammatory disease that usually results from abnormal activation of trypsin due to a variety of factors, which in turn causes self-digestion of pancreatic tissues, swelling, bleeding, and even tissue necrosis (4). Among them, acute pancreatitis (AP) is one of the common gastrointestinal diseases in hospitals with high mortality rate. AP is an acute inflammatory response of the pancreas in which the patient experiences sudden onset of severe epigastric pain accompanied by abdominal pressure pain, nausea, vomiting, fever, and increased pulse rate (5). And despite the low incidence of chronic pancreatitis (CP), it can cause permanent damage to the pancreas, which has a significant impact on the quality of life of patients (6). CP develops slowly, beginning with cellular injury followed by inflammation and fibrosis. The pathophysiologic process of chronic pancreatitis involves the alveolar cells, which are the primary site of initial injury or stress, leading to an inflammatory cascade response (7). Studies have revealed that local and systemic immune responses driven by inflammatory cells determine the course and severity of pancreatitis and are one of the important factors that induce pancreatitis (8). Several immune cells, including macrophages, granulocytes, dendritic cells (DCs), and T cells, play a crucial role in the pathologic process of pancreatitis (9).

The persistence of pancreatitis, especially CP, is considered an important risk factor for pancreatic cancer (PC) (10). The lethality of PC is so high that it ranks among the leading causes of cancer death worldwide (11). The tumor microenvironment (TME) exhibits immunosuppressive properties and is able to interfere with the effects of immunotherapy by promoting the recruitment of immunosuppressive cells and increasing the expression of immunosuppressive molecules (12). The TME of PC is a complex network of multiple cells and molecules, which includes PC cells, pancreatic stellate cells (PSCs), cancer-associated fibroblasts (CAFs), tumor-associated macrophages (TAMs), regulatory T cells (Tregs), extracellular matrix, vasculature, and lymphatic vessels, as well as a multitude of signaling molecules, such as growth factors, cytokines, and chemokines (13, 14). This microenvironment significantly affects PC progression and patient prognosis by enhancing fibrosis, promoting immune escape, and increasing treatment resistance. The IL-2 family, as an important player in the inflammatory response and a key factor in immune regulation, is tightly linked to the development of pancreatitis. Cytokines of the IL-2 family have been shown to influence the TME by modulating immune responses to affect TME, which in turn affects the growth and spread of tumor cells and participates in the development of PC.

Currently, there is no systematic review of the specific role of the IL-2 family in pancreatitis and PC. Therefore, in-depth studies in this area are of particular importance. In this paper, we will explore the structural features and functional properties of IL-2 family members and outline their roles and their molecular mechanisms in pancreatitis and PC, in order to deepen the understanding of the mechanisms of IL-2 family roles in pancreatic diseases and to lay a solid scientific foundation for the development of innovative therapeutic strategies for IL-2 family cytokines in pancreatic diseases.

## 2 IL-2 family

**Figure 1:**
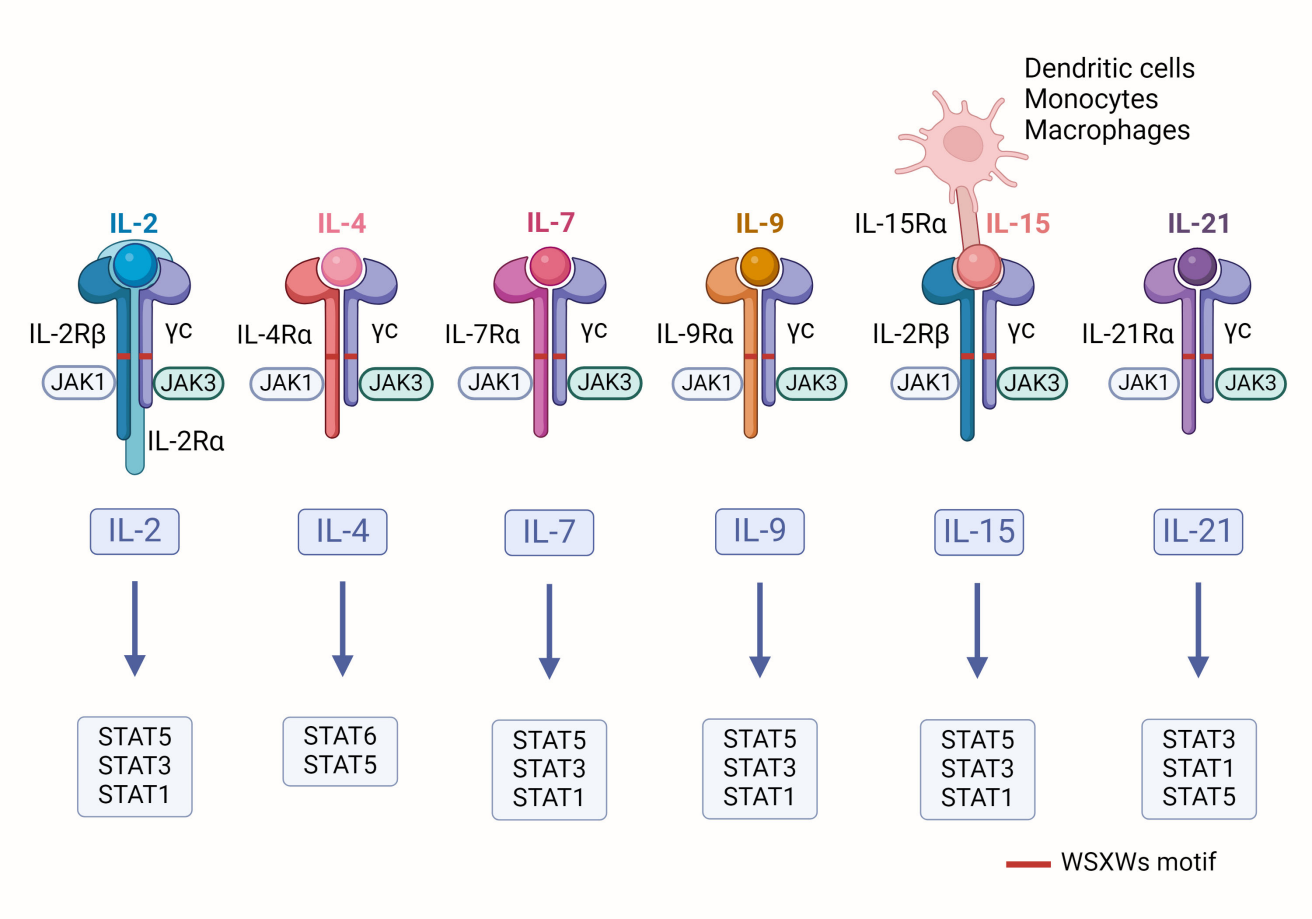
Schematic of IL-2 family cytokines: The receptors for IL-2 family cytokines consist of a common γ chain and their specific α chains. When these receptors bind to their corresponding cytokines, they initiate the activation of JAK kinases, which in turn trigger the phosphorylation of specific STAT proteins.

Interleukins (ILs) are proteins secreted by leukocytes that play crucial communicative roles in both the innate and adaptive immune systems, while also affecting the functions of non-immune cells and tissues (15). These molecules promote the proliferation, differentiation, and activation of immune cells, thereby enhancing the body’s immune response. Based on sequence similarity, secondary structure, receptor composition, and functional characteristics, scientists have classified over 60 types of ILs into seven different families (16). Among them, IL-2, IL-4, IL-7, IL-9, IL-15, and IL-21 are categorized as type I cytokines, collectively known as the IL-2 family, due to their similar three-dimensional structure characterized by a four α-helix bundle (17). These cytokines exert their effects by binding to specific receptors that typically consist of common γ chains and unique α chains (such as IL-2Rα, IL-4Rα, IL-7Rα, IL-9Rα, and IL-15Rα) (18), (as shown in **Figure 1**). There is a conserved sequence on these chains, the WSXWs motif, which plays a key role in the activation of the receptors. Therefore, the IL-2 family is sometimes referred to as the cytokine receptor γc family. Upon binding to their receptors, these cytokines can activate JAK1 and JAK3 kinases, leading to a cascade of phosphorylation reactions involving STAT proteins (such as STAT1, STAT3, STAT5, and STAT6) (19).

IL-2, the first member of the IL-2 family, was initially discovered in the supernatant of activated T cells and identified as a key factor for T cell growth (20). This 15 kDa cytokine is typically secreted by activated CD4+T cells and CD8+T cells (21). The IL-2 receptor complex is composed of three distinct subunits: IL-2Rα (CD25), IL-2Rβ (CD122), and IL-2Rγ (CD132). The primary function of IL-2 is to promote the proliferation and differentiation of effector T cells, memory T cells, and NK cells, thereby triggering immune responses (2). Additionally, IL-2 activates both T cells and B cells and regulates immune responses through activation-induced cell death (AICD) to maintain immune system balance. IL-2 is also crucial for the formation of Tregs and is essential for the function of Foxp3+ T cells in autoimmune diseases such as type 1 diabetes, systemic lupus erythematosus, pancreatitis, and pancreatic cancer (22).

IL-4 was initially identified as a pleiotropic type 2 cytokine, primarily secreted by CD4+T cells, Th2 cells, basophils, eosinophils, and mast cells (1). IL-4 can bind to its receptor, activating various signaling pathways, including JAK-STAT and PI3K/Akt/mTOR (23). IL-4 plays multiple roles in immune regulation: it promotes the survival and proliferation of mast cells, induces macrophage polarization towards the M2 phenotype (24), enhances B cell differentiation, facilitates immunoglobulin class switching, and increases the production of IgG and IgE (1).

Hunt and his colleagues first identified IL-7 as a cytokine that promotes the development of pro-B cells and early B cells (25). This active form is a 25 kDa single-chain glycoprotein, also known as lymphopoietin-1 (LP-1) (26). IL-7 plays a significant role in regulating the immune system by promoting the maturation of B and T cells, maintaining a reservoir of immature T cells, enhancing the proliferation of memory T cells, and increasing the cytotoxicity of NK cells (27, 28). Additionally, IL-7 is involved in the development and regulation of monocytes, macrophages, dendritic cells, and innate lymphoid cells (29).

Researchers identified a growth factor that promotes the sustained proliferation of human P40 T cells and named it IL-9 (30). This cytokine is a single-chain glycoprotein with a molecular weight of 32 to 39 kDa (30). IL-9 plays a crucial role in the immune system by promoting the proliferation of T cells, B cells, mast cells, and epithelial cells through the activation of multiple members of the STAT family, such as STAT1, STAT3, and STAT5 (31). Additionally, IL-9 contributes to the proliferation of other cell types, including erythroid progenitor cells, fetal thymocytes, Human megakaryocytic leukemia cell lines, and bone marrow progenitor cells (32).

IL-15 is a cytokine composed of 114 amino acids with a molecular weight of 14-15 kDa, known for stimulating T lymphocyte proliferation (33). Structurally, IL-15 is quite similar to IL-2, as they share the same receptor subunits, the β chain and γ chain, and can activate similar signaling pathways (34). Despite these structural and signaling similarities, IL-15 and IL-2 have distinct functions in the body. For instance, IL-15 can inhibit AICD triggered by IL-2 (35). IL-15 supports the survival of T cells, B cells, and NK cells by inhibiting apoptosis (36). It plays a critical role in the development, homeostasis, and function of NK cells, intraepithelial lymphocytes, dendritic cells, macrophages, and mast cells (37, 38). Additionally, IL-15 is involved in the generation and reactivation of naive T cells, effector T cells, and memory T cells (39).

Discovered in 2000, IL-21 is the most recently identified member of the IL-2 family (40). It is an autocrine cytokine primarily produced by follicular Th cells and Th17 cells, with a molecular weight of 15 kDa (41). IL-21 is notable for its potent regulatory effects on numerous immune cells. It enhances T cell expression and promotes Th cell development through the activation of JAK/STAT, MAPK, and PI3K pathways (42). Additionally, IL-21 is involved in the maturation and proliferation of B cells, class switching, and the production of antigen-specific antibodies (43, 44).

## 3 Pancreatic diseases

The development of pancreatitis is strongly associated with gallstones, hypertriglyceridemia and alcohol consumption (45). Under normal physiological conditions, zymogen is activated in the brush border of the small intestine, triggering the normal activation process of other zymogens. However, if zymogen is prematurely activated in the pancreas, it may cause inflammation, necrosis, and severe injury to pancreatic tissues, ultimately leading to the development of pancreatitis (46). Pancreatitis can be categorized into acute and chronic types based on its clinical manifestations and pathophysiological features. AP is an acute inflammatory response of the pancreas. Over the past decade, AP hospitalizations have increased by about 30%, costing the U.S. $2.6 billion annually in health care costs (47), and in the United States alone, approximately 300,000 people visit the emergency room each year for this condition (48). Patients with AP experience sudden onset of severe epigastric pain, which tends to radiate down the back and worsens after a meal, and is accompanied by abdominal compression pain, nausea, vomiting, fever and accelerated pulse rate (5). CP, on the other hand, is a persistent fibroinflammatory state caused by recurrent episodes of varying degrees of pancreatic inflammation, which ultimately leads to irreversible damage to the pancreatic tissue and loss of function (49). Compared with AP, the diagnosis of CP is more challenging, and its diagnostic criteria need to be further studied and clarified.

Abnormal immune cell infiltration and cytokine overexpression play a key role in the development of pancreatitis. Macrophages exhibit different phenotypes at different stages of AP and CP. In AP, they differentiate into the pro-inflammatory M1 type, whereas in CP, they gradually change from the initial M1 type to the M2 type with anti-inflammatory and pro-fibrotic properties (50). In CP, M1-type macrophages, as the main immune cells triggering the inflammatory response, promote the expression of pro-inflammatory factors such as TNF-α, TGF-α, and IL-6 (51), while M2-type macrophages secrete anti-inflammatory mediators such as TGF-β1 and IL-10 (52). It is well known that PSCs play an important role in pancreatic fibrosis by regulating the synthesis and degradation of extracellular matrices (e.g., fibronectin and type I/III collagen) (53). Interestingly, M2-type macrophages can interact with PSCs to stimulate the pro-pancreatic fibrosis effects of PSCs. M2-type macrophages activate PSCs by releasing the cytokines PDGF and TGF-β, and in turn activated PSCs secrete IL-4/IL-13 in an IL-4Ra-dependent manner, inducing macrophage polarization to the M2 phenotype (54).

**Figure 2:**
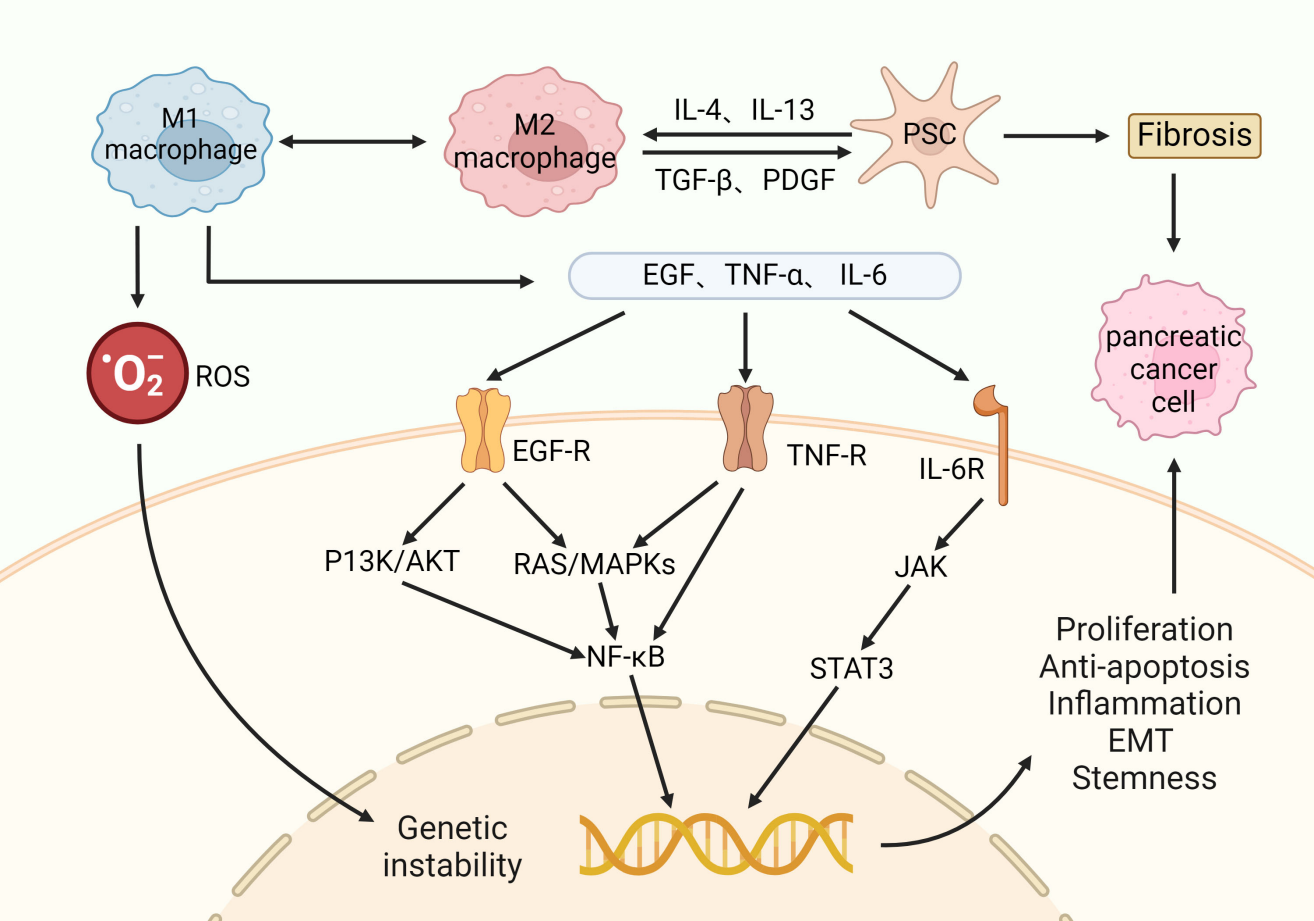
Macrophages in the progression of CP to PC: M2 macrophages interact with PSCs and contribute to the fibrotic process in the pancreas. At the same time, M1 macrophages release cytotoxins such as ROS, which promote genetic instability. M1 macrophages also release pro-inflammatory factors such as EGF, TNF-α, and IL-6, which bind to the corresponding receptors on the cell surface and activate multiple downstream oncogenic signaling pathways. Ultimately, CP cells exhibit proliferation, anti-apoptosis, inflammatory response, epithelial mesenchymal transition (EMT), and stem cell properties, completing its transformation into an invasive PC.

PC is an extremely lethal and aggressive malignant tumor whose global incidence has more than doubled in the last 25 years (55). The cure rate for PC is extremely low due to late diagnosis and limited treatment options (56). This has led to an increase in its mortality rate from fourth to third in the ranking of cancer causes of death in 2016 (11). A sustained inflammatory response is capable of forming pro-cancer TME and accelerating the transition from CP to PC. In particular, M1-type macrophages exacerbate inflammation and tissue damage by enhancing reactive oxygen species (ROS) production and triggering genetic instability (57). It also secretes a variety of pro-inflammatory cytokines, which bind to the corresponding receptors on the cell surface and subsequently activate signaling pathways such as PI3K/AKT, RAS/MAPK, and JAK, which further induce the activation of pro-proliferative and anti-apoptotic transcription factors such as NF-κB and STAT3 (58). Eventually, CP cells exhibit proliferation, anti-apoptosis, inflammatory response, epithelial mesenchymal transition (EMT), and stem cell properties, completing its transformation to invasive PC (as shown in **Figure 2**). In clinical practice, the diagnosis of PC usually requires a combination of symptoms, imaging tests and serum biomarkers. Its main symptoms include abdominal pain, abnormal liver function, jaundice, new-onset diabetes mellitus, dyspepsia, nausea and vomiting, back pain, and weight loss (59). CT angiography, magnetic resonance imaging, and ultrasound endoscopy are important imaging modalities (60). Carbohydrate antigen 19-9 is used as a biomarker with high sensitivity and specificity for the diagnosis of PC (61). PC cells activate immunosuppressive cells such as PSCs, myeloid-derived suppressor cells, TAMs, and Treg cells, which lead to immune escape from the tumor and exacerbate tumor progression and metastasis (10). Treatment of PC includes immunotherapy, surgery, and chemotherapy. In the treatment of PC, immunotherapy faces challenges due to factors such as fibrosis, hypoxia, and immunosuppressive properties of TME (62). However, immunotherapeutic studies for PC are still ongoing, and various aspects such as immune checkpoint blockade, vaccination, lysovirus, and overdose immunization are available (63). Therefore, continued research into the immune mechanisms of PC is crucial for future prevention and treatment strategies.

## 4 IL-2 family and pancreatic diseases

The IL-2 family plays a crucial role in immune responses by regulating immune cells such as T cells and NK cells, significantly impacting immune-mediated diseases. As our understanding of pancreatic function and its role in diseases deepens, it becomes clear that immune regulation is vital in the development of pancreatitis and PC. Therefore, investigating the potential link between the IL-2 family and these pancreatic diseases is essential for comprehending their immunological background and providing a scientific basis for the development of prevention, diagnosis, and treatment strategies.

### 4.1 IL-2

**Figure 3:**
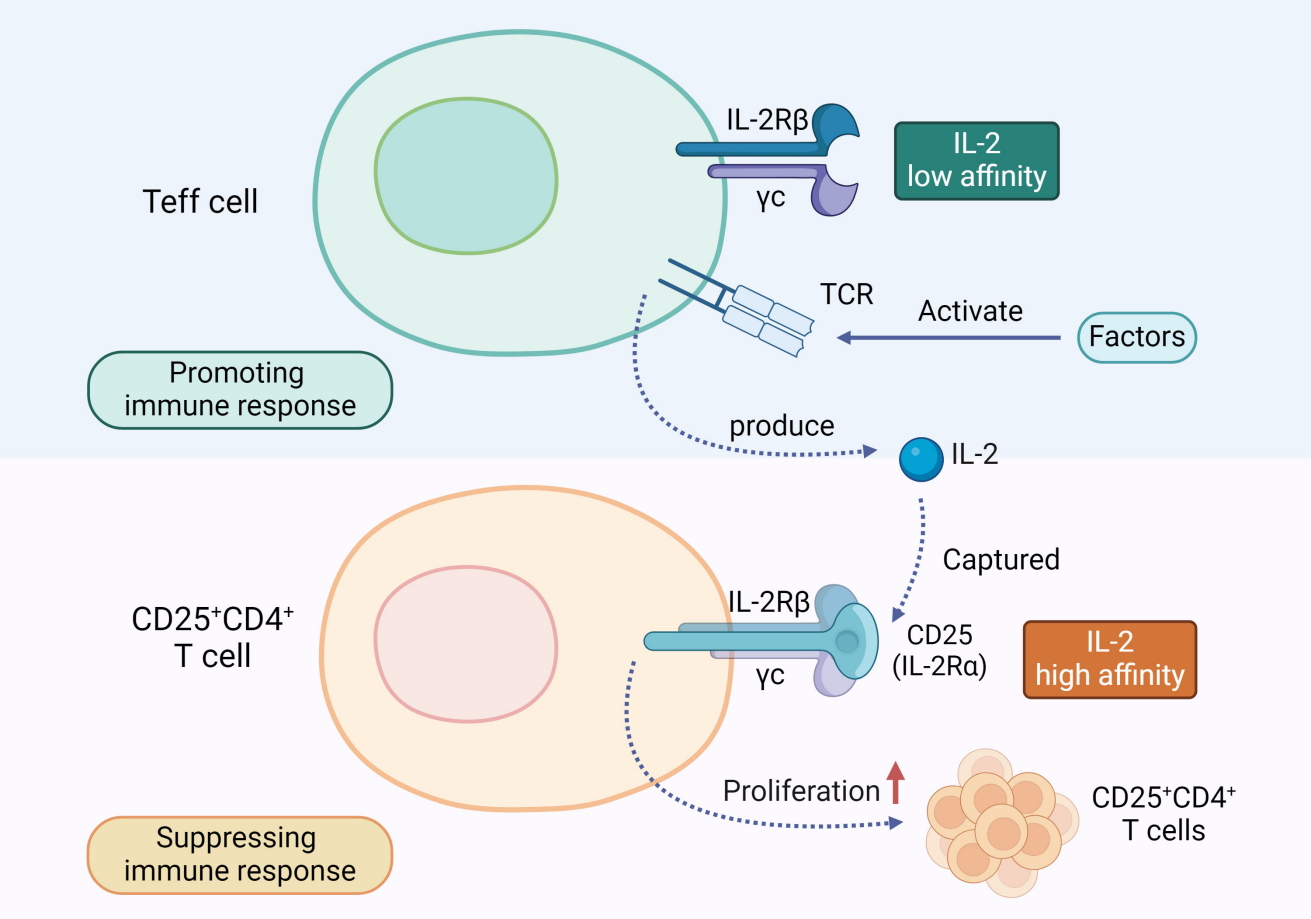
IL-2 and T Cell Regulation Mechanism: Teff cells and CD25+CD4+T cells have different types of receptors on their surfaces. When the TCR on Teff cells is activated, it prompts the Teff cells to release IL-2. This IL-2 is preferentially captured by the high-affinity IL-2 receptor CD25 on the surface of CD25+CD4+T cells, promoting their proliferation and exerting an immunosuppressive function.

In pancreatitis, IL-2 acts as a key immunoregulatory factor, significantly promoting the proliferation of Tregs, especially the CD25+CD4+T cell subset. Tregs suppress excessive immune responses through various complex mechanisms, including the release of anti-inflammatory cytokines like TGF-β, IL-10, and IL-35; hydrolyzing ATP into immunosuppressive adenosine; blocking stimulatory signals of CD80 and CD86; and directly eliminating inflammatory cells with cytotoxic molecules (64). Early studies found that knocking out IL-2 (65), IL-2Rβ (66), or CD25 (67) genes in mice leads to systemic inflammation and abnormal lymphocyte proliferation. Specifically, the absence of CD25 results in Treg apoptosis, triggering fatal autoimmune responses. Similar conclusions have been drawn from human studies, where disrupting the expression of CD25 and IL-2Rβ genes reduces their *in vitro* inhibitory capacity, causing autoimmune inflammatory diseases (68, 69). This is because, in the absence of IL-2 signaling, Treg proliferation is inhibited. Although Treg cells cannot produce IL-2, they have high-affinity CD25 on their surface, enabling them to capture IL-2 during immune responses, ensuring their survival. When IL-2 is lacking, Treg suppression is impaired, disrupting immune homeostasis and leading to autoimmune diseases like pancreatitis. Effector T cells (Teff cells), on the other hand, only have low-affinity IL-2 receptors (IL-2Rβ and IL-2Rγ) on their surface, which prevents them from binding IL-2 preferentially, thus limiting their immune activation (70). However, when T-cell receptors (TCRs) are stimulated, Teff cells can produce IL-2 (71). Concurrently, IL-2 is preferentially captured by CD25 on Treg cells, promoting Treg proliferation through IL-2R binding. In AP, IL-2 employs these mechanisms to exert its immunosuppressive effects, preventing excessive immune responses (as illustrated in **Figure 3**). Therefore, IL-2 not only helps maintain immune homeostasis but also potentially prevents the worsening of AP and its progression to CP.

In CP, IL-2 has a dual role. On one hand, the aforementioned anti-inflammatory mechanism of IL-2 also applies to CP, helping to reduce pancreatic fibrosis. By promoting Treg growth, IL-2 can inhibit Th2 cells and type 2 innate lymphoid cells in the type 2 immune response, thereby reducing the expression of pro-fibrotic cytokines and helping to control the progression of pancreatic fibrosis (72). On the other hand, excessive activation of IL-2 signaling may exacerbate the inflammatory response, leading to further damage to pancreatic tissue. For instance, IL-2 can increase the production of pro-inflammatory cytokines such as TNF-α and IL-1β and promote the infiltration of immune cells into pancreatic tissue, worsening inflammation and tissue damage (73).

In PC, the function of IL-2 is equally complex. On one hand, IL-2 can enhance the immune response against tumors by activating Treg cells. By inducing T cell expression of CD25, IL-2 enhances T cell signaling, thereby increasing the anti-tumor efficacy of cytotoxic T-lymphocytes (CTL) and chimeric antigen receptor T cells (CAR-T) used in immunotherapy (74). In pancreatic ductal adenocarcinoma (PDAC), increased levels of IL-2 correlate with improved patient survival and enhance anti-tumor immunity, particularly by increasing CD8+ T cells, B cells, and NK cells (75). Although IL-2 does not act directly on dendritic cells (DCs), it can indirectly promote the expansion and activation of DCs through activation of T-cells and NK-cells, which can lead to an improvement in anti-tumor immune response (76). In addition, IL-2 treatment improves TME by promoting tumor infiltration by DCs and enhances sustained T-cell immunity against PDAC, whereas co-culture of IL-2 with PBMC significantly enhances the anti-tumor effects of DCs (75). A separate study demonstrated that IL-2 not only facilitates the *in vitro* expansion of tumor-infiltrating lymphocytes (TIL) and enhances the generation of CD8-positive TIL, thereby improving their tumor recognition and attack capabilities (77). Additionally, IL-2 is used in conjunction with agonist anti-CD3 and 4-1BB antibodies to activate and strengthen the patient’s immune system, particularly T cells, which in turn amplifies immune responses against PDAC (78). Sun and other scientists have designed a recombinant IL-2 immune cytokine combining tumor-localizing antibodies and super mutant IL-2 (sumIL-2), which specifically binds to tumor cells and cytotoxic T cells (CTLs), thus effectively enhancing the anti-cancer effect (79). Moreover, preoperative treatment with sumIL-2 helps prolong patient survival compared to conventional adjuvant therapy.

On the other hand, IL-2 can promote the development of PC by affecting the TME. Tumor cells exploit the IL-2 signaling pathway to promote their proliferation and survival or alter the function of immune cells to evade immune surveillance. The IL-2-STAT5 signaling pathway is the central mechanism of this immune response, promoting T-cell proliferation and survival and regulating the immune response to prevent autoimmune diseases. The pathway activates JAK kinase and STAT5 by binding IL-2 to its receptor, and then phosphorylated STAT5 enters the nucleus to regulate gene expression (80). In addition, IL-2 is involved in the establishment of anti-tumor immune responses and immune tolerance, plays a crucial role in T-cell development and function, and is central to the progression of a variety of immune-related diseases. In the case of PCs, for example, high expression of pro-inflammatory cytokine-IL-18 is strongly associated with advanced pathological staging and poor patient prognosis.IL-18 leads to CD8+ T-cell depletion by enhancing IL-2R signaling and activating JAK kinase and p-STAT5 (81). This process not only weakens the immune attack against the tumor, but also reduces the efficacy of immune surveillance, interferes with the formation of memory T-cells, enhances immunosuppression within the TME, inhibits T-cell proliferation and survival, reduces the diversity of T-cell receptors, promotes the escape of the tumor from immune attack, and may affect the efficacy of immunotherapy (82). Additionally, IL-2 can polarize TAMs to the M2 phenotype, aiding tumor cells in evading immune surveillance and promoting tumor growth and metastasis (83). Research also indicates that abnormal activation of the IL-2 signaling pathway may be associated with chemotherapy resistance in PC. To address the issue of radiotherapy (RT) resistance in PDAC, researchers have developed a novel PD-1 targeted IL-2 variant called PD1-IL2v (84). This variant selectively binds to CD122 and CD132 without binding to CD25, preventing excessive systemic spread of IL-2 and reducing potential side effects (84). PD1-IL2v specifically binds to PD-1 receptors on effector immune cells, promoting CD8+T cell proliferation and inhibiting tumor growth and metastasis (85). Additionally, PD1-IL2v reduces Treg production while continuously generating effector T cells, further inhibiting tumor development and spread (85). When combined with RT, PD1-IL2v exhibits sustained and synergistic anti-tumor effects, significantly reducing PDAC treatment resistance. The combination of recombinant IL-2 and allicin significantly increased the proportions of CD4+, CD8+ T cells and NK cells in the peripheral blood of mice and inhibited PCs by 90.5% (86). These strategies offer new possibilities for improving cancer treatment outcomes.

In summary, IL-2 plays multiple roles in immune regulation and is commonly used in clinical treatments for inflammation and tumors. IL-2 is a key cytokine for the differentiation and activation of cytotoxic effector cells (87). To avoid severe side effects that can arise from systemic IL-2 therapy, lower doses of IL-2 are often used in clinical settings. This approach takes advantage of the high sensitivity of Tregs to IL-2, promoting immune tolerance by increasing Treg numbers (88). However, a decrease in IL-2 levels does not always indicate improvement and may signal worsening pain. In CP, for instance, patients with severe pain have lower detectable levels of IL-2 compared to those with mild or no pain (89), possibly due to IL-2’s antinociceptive effects (90). Despite the limitations and potential adverse reactions associated with high-dose IL-2, such as the activation of a large number of Tregs, its use is still necessary in certain cases (3). For example, high-dose IL-2 binding to CD25 can enhance IL-2R signaling in CD4+and CD8+T cells in mice, improving the efficacy of vaccines against antigens and more effectively clearing tumor cells (91). Therefore, precise modulation of IL-2’s immunoregulatory effects is essential in treating pancreatitis and PC to alleviate symptoms and slow disease progression.

### 4.2 IL-4

In AP, various traditional Chinese medicines have been shown to alleviate symptoms by enhancing IL-4 expression. For instance, Da Cheng Qi Tang, a herbal formula containing rhubarb, magnolia bark, bitter orange, and mirabilite, boosts IL-4 production and exerts anti-inflammatory effects (92). Baicalin, a flavonoid extracted from the dried root of Scutellaria baicalensis, not only promotes IL-4 expression but also reduces pyroptosis in pancreatic alveolar cells (PACs), alleviating tissue damage and inflammation in hyperlipidemic pancreatitis (93). In models of severe acute pancreatitis (SAP) with lung injury, IL-4 promotes the shift of lung macrophages from a pro-inflammatory M1 type to an anti-inflammatory M2 type, reducing inflammation associated with SAP (94). In SAP mouse models, intraperitoneal injection of IL-4 lowers levels of complement regulatory factors DAF and CD59 in the pancreas, significantly improving pancreatic necrosis (95). IL-4 displays its anti-inflammatory properties through various mechanisms: inhibiting macrophage activation and accumulation (96), preventing the production of pro-inflammatory cytokines TNF-α and IL-1β (97), upregulating the expression of IL-1 receptor antagonist (98), and activating macrophage 15-lipoxygenase (99). These actions may explain the molecular mechanisms by which IL-4 improves SAP and related inflammation.

In contrast to AP, IL-4 may promote disease progression in CP. Studies have shown that elevated IL-4 levels in CP mouse models lead to macrophage polarization to the M2 type, exacerbating pancreatic fibrosis (100). Thioredoxin 1, a redox balance regulator, can inactivate IL-4 by reducing its C46-C99 disulfide bond, thereby inhibiting the generation of M2 macrophages, reducing inflammation, and slowing fibrosis progression in CP (100). Notably, there may be a positive feedback mechanism involving IL-4, PSCs, and macrophages that promotes pancreatic fibrosis in CP. IL-4 released by PSCs promotes the alternative activation of macrophages (54), which in turn produce IL-4R ligands that further activate PSCs. However, IL-4 transfer to PSCs is accompanied by increased expression of the anti-inflammatory cytokine IL-10, helping to suppress inflammation and fibrosis (101). Therefore, the specific role of IL-4 in CP requires further research and exploration.

In the TME of PC, IL-4 levels are elevated, promoting cancer cell growth and acting as an immunosuppressive factor, thereby limiting the effectiveness of immunotherapy. Studies have found that plasma IL-4 levels in PDAC patients are significantly higher than in healthy individuals, and high IL-4 levels are associated with poor prognosis (102). IL-4 promotes PC progression through various mechanisms, including enhancing the activity of cathepsins B and S in TAMs (103), inducing M2-type TAM polarization (104), and activating STAT transcription factors and related signaling pathways (105), which inhibit T cell immune responses against PC. In PC cells, the expression levels of the transcription factors STAT1, STAT3, and STAT6 differed, with the phosphorylation of STAT3 enhanced by IL-4 stimulation (105) and the nuclear translocation of STAT6 increased in response to IL-4 (106). Dual oxidase 2 (DUOX2) is a key glycoprotein that regulates cellular redox and is associated with chronic inflammation and tumor development (107). In PDAC, upregulation of IL-4 promotes nuclear translocation of STAT6 and significantly increases the expression of DUOX2 mRNA and protein, which drives the development of PDAC and enhances its invasiveness and migration (106). Studies have shown that increased expression of IRS-2 in human PC may promote tumor mitosis and growth through activation of the PI3K signaling pathway (108). Researchers such as Prokopchuk et al. found that IL-4 was able to elicit enhanced tyrosine phosphorylation of IRS-1 and IRS-2, as well as increase the activity of the MAPK and PI3K/Akt signaling pathways (105). In addition, the research team of Traub et al. found that exogenous stimulation of IL-4 could lead to enhanced phosphorylation of pro-oncogenic signals such as c-Jun, ERK-1/2, and STAT3 in Capan1 cells (109), and that the specific STAT3 phosphorylation inhibitor LLL12 was able to inhibit the survival of PC cells (110). Additionally, IL-4 weakens antigen-dependent T cell proliferation, increases T cell exhaustion, and reduces cytolytic capacity. Cell surface receptors, especially IL-4Rα, are highly specific targets in PC therapy and can directly kill tumor cells overexpressing these receptors while reducing damage to normal tissues. In PC, overexpression of IL-4Rα attenuates cell growth and migration after its expression is reduced by shRNA plasmids, and impaired IL-4 signaling in PC cells enhances the inhibition of xenograft tumors (109), suggesting that IL-4Rα is a promising therapeutic target.

Currently, CAR-T technology has proven effective in overcoming IL-4-induced immunosuppression and has become a promising option for cancer treatment. For example, the inverted cytokine receptor (ICR) of IL-4/IL-7, composed of the IL-4R extracellular domain and the IL-7R intracellular domain, reduces IL-4’s inhibitory effect on CAR-T cells, promoting T cell proliferation and enhancing anti-tumor activity (111). Other types of CAR-T cells, such as 4/15NKG2D-CAR and IL-4/IL-21 ICR, also show anti-tumor activity; the former converts IL-4 inhibitory signals downstream into IL-15 activation signals (112), while the latter activates the STAT3 pathway (113). Therefore, gene-engineered therapies related to IL-4 hold promise as new treatments for PC. However, research by Seifert, M., et al. found that adding IL-4 to anti-CD40 monoclonal antibodies inhibits IgM reactivity to tumor antigens while enhancing B cell immune responses to PC antigens, aiding the immune system in recognizing and eliminating PC cells (114). This indicates that IL-4 may exert anti-tumor activity by affecting the proliferation, apoptosis, and migration of PC cells. Recombinant IL-4-PE toxins, such as IL-4-PE38QQR (115) and IL4(38-37)-PE38KDEL (116), have been shown to inhibit PC progression. Another cytotoxin, consisting of IL-4 and truncated Pseudomonas exotoxin, has specific cytotoxicity against PC cells and demonstrates synergistic antitumor effects *in vitro* and in various mouse models when combined with gemcitabine (117). Molecules targeting cytokine receptors may have significant toxicity in cancer therapy. In addition, a hybrid peptide (IL-4Rα-lytic) and cytotoxic cleavage peptide, which bind IL-4Rα, showed anticancer effects in PC cells expressing IL-4Rα and in xenograft mouse models (118). These findings further confirm the potential of IL-4Rα and its related pathways in PC therapy. In addition, blocking IL-4 had a significant inhibitory effect on PC development, and in particular, the use of IL-4-neutralizing antibodies was able to inhibit the basal proliferation of COLO-357, PANC-1, and MIA PaCa-2 cell lines (105). More critically, by inhibiting IL-4 mRNA expression in the livers of patients with PC malignancy (119) and by improving the physical condition of IL-4-treated tumor-bearing mice (120), it may provide a potentially effective approach for the future treatment of PC patients with tumor-induced malignancy.

In summary, in AP, IL-4 primarily exhibits anti-inflammatory properties and promotes tissue repair. In contrast, in CP and PC, while some evidence suggests that IL-4 may inhibit disease progression, most studies support the notion that IL-4 promotes disease development.

### 4.3 IL-7

IL-7 has been shown to play a role in the progression of pancreatic diseases. In pancreatitis, IL-7 expression levels increase over time, peaking within 72 hours of the acute phase, indicating its involvement in the inflammatory response of pancreatitis (121). In CP, IL-7 can activate fibroblasts, thereby exacerbating pancreatic fibrosis (122). While the exact mechanisms of IL-7 in pancreatitis are not yet well-defined, it is known that IL-7 regulates T cell function and is involved in various immune-related diseases, including diabetes and PC (123, 124). For instance, in type 1 diabetes, IL-7 promotes T cell proliferation by binding to its receptor and activating the JAK1, JAK3, and STAT5 signaling pathways, potentially worsening the disease (123). Future research could further explore the mechanisms of IL-7’s interaction with T cells in pancreatitis.

**Figure 4:**
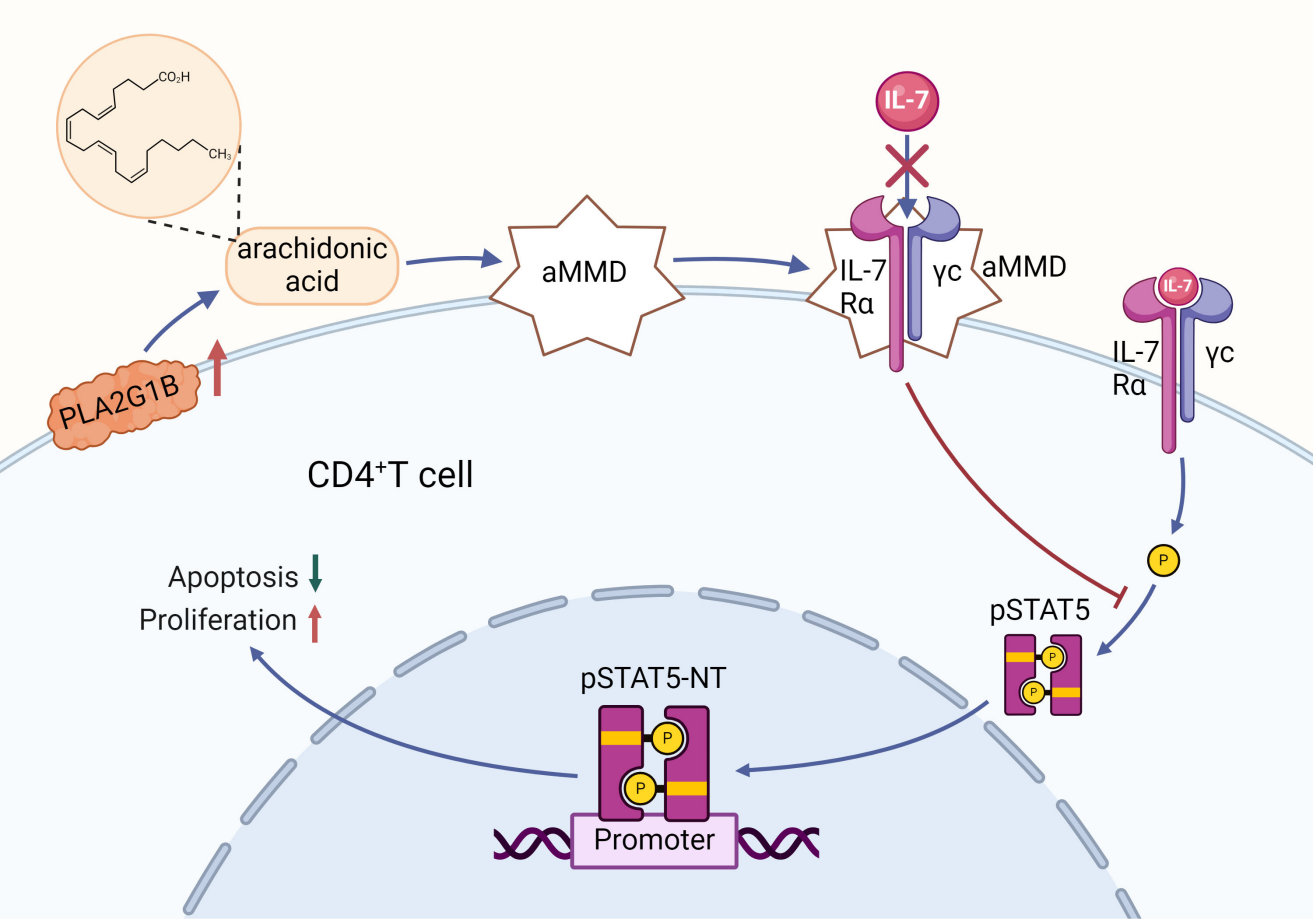
Regulation of CD4+ T cell proliferation by the IL-7 signaling pathway and its mechanism of PLA2G1B-mediated inhibition: Binding of IL-7 to its receptor triggers the phosphorylation of STAT5 (pSTAT5), a process that facilitates the translocation of pSTAT5 to the nucleus (NT), contributing to the reduction of apoptosis and the promotion of proliferation in CD4+ T cells. However, an increase in PLA2G1B leads to the release of arachidonic acid (AA), which in turn contributes to the formation of abnormal membrane microdomains (aMMDs). These aMMDs are capable of intercepting the IL-7 receptor and preventing its interaction with IL-7, which in turn inhibits pSTAT5 activation and nuclear translocation, adversely affecting the survival and proliferation of CD4+ T cells.

In the field of PC research, the role of IL-7 has been partially elucidated, with some studies suggesting that IL-7 could serve as a potential biomarker for diagnosing PC (125–127). Jang’s study found that IL-7R and its mRNA levels in PDAC patients were significantly higher than those in patients with AP and CP (125). Heo’s research indicated that IL-7R can be detected in the early stages of PDAC formation, potentially aiding doctors in providing earlier immunotherapy strategies to improve patient survival rates (126). Sahar’s study showed that serum IL-7 levels in patients with autoimmune pancreatitis were significantly higher than in PDAC patients, which is important for clinical diagnosis and treatment decisions (127). IL-7 not only serves as a biomarker but also promotes PC development by affecting the apoptosis and maturation of immune cells. IL-7 binds to IL-7Rα, triggering activation of IL-7Rα-related tyrosine kinases, JAK 1 and JAK3. Activated JAK proteins phosphorylate specific motifs on the IL-7Rα chain to form a binding site for STAT5, which is then bound and phosphorylated to form a dimer and enter the nucleus. In this process, a range of genes in the nucleus that regulate cell growth and survival are affected, other pathways such as PI3K-AKT and MEK/ERK are activated, and the anti-apoptotic proteins Bcl-XL, Bcl-2, and MCL-1 are upregulated, whereas the pro-apoptotic proteins BAX and BAD are downregulated, resulting in increased T cell survival *in vivo* (128). IL-7 signaling can maintain the survival of memory CD8 T cells by mediating STAT5 and STAT3 activation (129). IL-7 not only activates STAT5 and thus promotes T cell proliferation, differentiation and survival, but also regulates T cell cytotoxicity and drug resistance (130). In PDAC, IL-7 and its activation of the JAK/STAT signaling pathway may make tumor cells resistant to chemotherapeutic agents such as gemcitabine and accelerate tumor progression (131).Normally, IL-7 binding to its receptor promotes the growth and maturation of CD4+T cells by facilitating pSTAT5-NT (132). However, in an immune-deficient environment, secretory proteases like Phospholipase A2 group 1B (PLA2G1B) located on the CD4+T cell membrane may release arachidonic acid (AA), forming abnormal membrane microdomains (aMMDs) on the cell membrane (133). These aMMDs can capture IL-7Rα and γc chains, disrupting normal IL-7 receptor binding and leading to elevated plasma IL-7 levels in PDAC patients (133). High IL-7 levels inhibit STAT5 phosphorylation and nuclear translocation, reducing CD4+T cell proliferation and impairing their anti-tumor immune function, thereby promoting PDAC development (134). This may explain the pro-carcinogenic mechanism of IL-7 in the TME (as illustrated in **Figure 4**). Given the high expression of IL-7 in PC, researchers have developed a novel CAR-T cell therapy centered around IL-7, capable of secreting human IL-7 and CCL19. This design not only alleviates the issue of T cell infiltration in tumors but also enhances CAR-T cell survival, showing significant anti-tumor effects in PC treatment (135). Li and other investigators (136) developed a novel drug, IL-7-Fc fusion protein (NT-I7), which enhances therapeutic efficacy through enhanced proliferation, activation, and prolonged survival of CAR-T cells.NT-I7 was able to significantly boost the number of CD4+ CAR-T cells, reduce the level of depletion markers on T cells, and enhance T cell persistence and tumor penetration. The development of each of these drugs offers new strategies and hope for PC treatment.

### 4.4 IL-9

IL-9 plays a crucial role in pancreatic diseases. The activation of cathepsin B triggers the activation of trypsin, which is a key step in the onset of AP (137). Xiao et al. found that mogroside IIE, a traditional Chinese medicine component used to treat PC, can reduce cathepsin B activity (138). Their experimental results indicated that mogroside IIE can inhibit the release of IL-9 in mice with AP (138). When exogenous IL-9 was injected into the mice, the effect of mogroside IIE in reducing the activities of cathepsin B and trypsin was negated, suggesting that IL-9 is essential for activating cathepsin B in the pancreas. Further experiments showed that administering an IL-9R antibody to the model could neutralize the effects of IL-9, restoring the activity of mogroside IIE and improving AP (138). These findings support the hypothesis that the pathogenesis of AP may be related to the IL-9/IL-9R signaling pathway. However, current research on the role of IL-9 in pancreatitis is still insufficient, and more studies are needed to further explore and validate the specific mechanisms of IL-9’s action.

In PC research, the role of IL-9 remains controversial despite considerable attention. Hu et al. suggested that IL-9 may promote the proliferation and metastasis of PC cells (139). Lu’s experiments showed that targeting and blocking IL-9 could effectively inhibit tumor growth in a PC mouse model (140). However, Th9 cells, a major subset of CD4+T cells, have been shown to produce high levels of IL-9 and exert anti-tumor effects in certain solid tumors such as breast cancer (141) and lung cancer (142). In the TME of PC, Th9 cells are the primary source of IL-9 (143), which suggests that IL-9 should have anti-tumor functions. Chen’s research indicated that Th9 cells can activate the cytolytic and secretory functions of NK cells, thus playing a role in tumor surveillance and inhibiting PDAC growth (144). Ahmed’s study also found that high levels of IL-9 expression in the PDAC stroma are associated with better prognosis, though IL-9 levels in tumor epithelium and serum showed no significant correlation with patient survival rates (143). Given the complex nature of IL-9 in PC, it has become a potential target for gene-engineered T cell therapy. The IL-9 signaling pathway is not typically a significant player in T cell activation due to the scant expression of IL-9R on T cell surfaces (145). Nevertheless, researchers have ingeniously tapped into this pathway for T cell application by engineering the chimeric receptor o9R (146). This receptor is composed of the extracellular domain (ECD) of the IL-2 receptor and the intracellular domain (ICD) of the IL-9 receptor, which interacts with modified IL-2 (oIL-2), initiating JAK activation and the subsequent phosphorylation of the crucial transcription factors STAT1, STAT3, and STAT5. These phosphorylated factors then dimerize and migrate to the nucleus to modulate gene expression, influencing cell proliferation, differentiation, and survival. The o9R signaling in T cells simultaneously activates STAT1, STAT3, and STAT5, a rare event in natural IL-9 signaling, which may augment T cell stem cell memory characteristics and effector functions. T cells equipped with o9R signaling have shown enhanced antitumor efficacy in both *in vitro* and *in vivo* models, marked by increased T cell expansion, viability, and tumor infiltration capabilities. This breakthrough is poised to mitigate T cell exhaustion and surmount the immunosuppression encountered in solid tumor therapies, including those for PC.

### 4.5 IL-15

IL-15 is closely linked to the progression of pancreatitis, with its serum levels serving as a predictive marker for AP. Studies have shown that serum IL-15 levels in patients with mild AP are lower than in healthy individuals, which may be a protective mechanism by the body to limit inflammation (147). However, in SAP patients, IL-15 levels are significantly elevated and are associated with disease severity, organ dysfunction, infection risk, and poor prognosis (147). Lower serum IL-15 levels generally indicate a better prognosis, while higher levels may suggest a worse outcome. Additionally, IL-15 levels are negatively correlated with CD4+T and CD8+T cell counts, indicating that it may affect T cell proliferation (147). In a study by Saito et al., IL-15 inhibited the apoptosis of CD4+and CD8+T cells in a rat model of sepsis, improved immune suppression caused by T cell exhaustion, and enhanced the immune response to pathogens, potentially helping to prevent secondary infections (148). This suggests that the regulatory relationship between IL-15 and T cells may be influenced by the tissue environment and pathogenic factors, warranting further investigation. IL-15 exerts a range of therapeutic effects in CP through diverse mechanisms: it mitigates fibrosis by alleviating islet cell atrophy, diminishing collagen accumulation, and suppressing the expression of genes associated with fibrosis. Additionally, it modulates immunity, safeguarding the pancreas by bolstering iNKT cell counts in the blood and stimulating their production of IFN-γ. IL-15 also engages the STAT signaling pathway by binding to IL-15Rα, notably activating STAT5, which modulates the activity of NKT cells (149). Furthermore, it curbs inflammatory responses and limits the influx of inflammatory cells. Directly targeting PSCs, IL-15 curbs their activation and proliferation. It also advances the apoptosis of immune and tumor cells and governs autophagy, thereby impacting cellular survival and functionality (149). These combined effects make IL-15 a potential therapeutic tool for improving CP pathology. Another experiment confirmed these mechanisms and demonstrated that IL-15 also improved CP-related acute lung injury (150). Thus, IL-15 presents a potential immunotherapeutic approach for CP patients, offering new treatment strategies, particularly in the context of fibrosis and malignant phenotypes.

IL-15 exerts multiple mechanisms of action in the treatment of CP: it exhibits anti-fibrotic effects, which include attenuating islet cell atrophy, decreasing collagen deposition, and inhibiting the expression of fibrosis-related genes. In addition, IL-15 enhances immune protection by elevating the number of iNKT cells in the blood and stimulating the production of IFN-γ by these cells. It also activates STAT family signaling, particularly STAT5, by binding to IL-15Rα, which in turn regulates NKT cell activity.IL-15 also inhibits inflammatory responses and reduces the accumulation of inflammatory cells. It acts directly on PSCs, inhibiting the activation and proliferation of these cells. In addition, IL-15 promotes apoptosis in immune cells and tumor cells and regulates the autophagic process, thereby affecting cell survival and function. These interacting mechanisms confer potential therapeutic value to IL-15 in ameliorating CP pathology.

Research on IL-15 in PC has garnered extensive attention. IL-15 is not only negatively correlated with the progression of PDAC but is also considered an important independent predictor of metastatic PDAC. In the South African population, IL-15 has been identified as a potential prognostic biomarker for metastatic disease in PDAC patients (151). IL-15 shows potential in PC treatment by promoting the generation, proliferation, and activity of anti-tumor NK cells and CD8+T cells, thereby enhancing the immune response against tumors (152). Although clinical studies have indicated that IL-15 has potential effects when combined with existing drugs, its efficacy as a monotherapy is limited due to its short half-life (153). Consequently, IL-15-based combination therapies are being widely researched. The combination of IL-15 and metformin showed excellent efficacy in PC therapy, which significantly inhibited the expression of the cell cycle-promoting Cyclin D1 and the anti-apoptotic protein Bcl-2 and promoted the up-regulation of the expression of the apoptosis-promoting protein Bax in tumor cells (154). In addition, since overactivation of the Akt/mTOR/STAT3 signaling pathway inhibits autophagy, the combination therapy, by inhibiting this signaling pathway, deregulated the inhibition of autophagy and enhanced the expression of the autophagy proteins LC3 and Beclin-1. Together, these changes at the molecular level promoted autophagy and apoptosis in tumor cells, demonstrating its anti-tumor potential (154). The Van team discovered that the combined use of CD40 agonists and IL-15 can enhance immune response and anti-cancer effects (155). Their experiments showed that the combination therapy enhances the efficiency of IL-15 binding to CD8+T cells and NK cells by promoting IL-15Rα expression through the CD40 agonist, resulting in a synergistic effect and significantly improving the long-term survival rates of PDAC mice (155). Utilizing the more stable binding characteristic of IL-15 with IL-15Rα, four IL-15 superagonists (composed of IL-15 and truncated forms of IL-15Rα complexes) have been designed and entered clinical development, yielding encouraging preliminary results (156). This opens new prospects for the future clinical treatment of malignant tumors such as PC.

Bioengineering based on IL-15 has opened a new avenue for the treatment of PC, providing an alternative to traditional combination therapies. For example, hIL15-ABD, a fusion of IL-15 with the albumin-binding domain (ABD), has been used to treat PDAC, enhancing anti-tumor effects and extending the drug’s half-life (157). When combined with the chemotherapy drug Nab-paclitaxel, hIL15-ABD effectively reduces the number of immunosuppressive Tregs, while promoting the accumulation of CD8+T cells, NK cells, and M1-type macrophages, significantly inhibiting PDAC growth in mice (157). Nelson et al. developed an oncolytic virus, VSV-IL-15, which enhances anti-tumor immune responses by transducing IL-15, increasing PDAC regression rates and extending patient survival (158). Additionally, a triple therapy combining NK cell immunotherapy, VSV-IL-15 virotherapy, and PD-1 blockade further strengthened immune cell activation and function, leading to more tumor regressions, with some patients achieving complete tumor eradication (158). Zhao et al. developed a dual cross-linked hydrogel system, P-aTIGIT&PDA@IL-15@gel, capable of sustained release of IL-15 and anti-TIGIT, effectively reactivating CD8+T cells and NK cells, and inhibiting residual tumor growth and distant metastasis in mouse PC models (159). Similar to previously mentioned factors, many IL-15-based genetically engineered T cells have been designed. In PDAC, cancer cells and CAFs within the TME jointly promote tumor growth and metastasis. Astrid and her team developed CD70-CAR-IL-15 NK cells, which demonstrated the ability to eliminate CAFs and improve prognosis, particularly in PDAC patients with significant desmoplasia (160). By adding IL-15 to CAR-iMacs targeting prostate stem cell antigen (PSCA), Shah et al. not only enhanced the viability of these macrophages to avoid apoptosis, but also succeeded in activating NK cells and T cells, which in turn boosted adaptive immune responses to PC (161). Teng et al. designed PSCA CAR_s15 NK cells, which enhance IL-15 activation of NK cells, improving tumor cell killing efficiency and showing significant inhibitory effects on PSCA-positive PC cells (162). Xu and his team developed a novel CAR-NK92 cell line, incorporating the IL-15Rα-sushi/IL-15 complex and a PD-1 signal inverter, named SP (Sushi-IL15-PD1) (163). This cell line targets PD-L1-expressing tumor cells, enhancing cytotoxicity and exhibiting significant killing effects on PDAC cells (163). These studies bring innovative perspectives and approaches to PC treatment, highlighting the need for ongoing research and development in this field.

Interestingly, exercise can stimulate the release of IL-15 from skeletal muscle, prompting researchers to explore the potential of exercise-regulated IL-15 levels for treating PC. Kurz et al. found that aerobic exercise activates the IL-15/IL-15Rα pathway, promoting CD8+T cell infiltration into PDAC cells, thereby enhancing anti-tumor immune responses in both mice and humans (164). Additionally, using IL-15 superagonists to activate this pathway can mimic the anti-tumor effects of exercise, including tumor growth inhibition, prolonged survival, and enhanced chemotherapy efficacy, providing a new theoretical basis for PC treatment strategies (164). Moderate exercise has also been found to increase IL-15Rα levels in adipose tissue, reducing the risk of obesity-related PDAC (165). In non-obese mice, overexpression of IL-15 helps slow PDAC progression, while exercise itself may counteract obesity-induced PDAC progression by reducing inflammation (165). These findings further highlight the potential value of exercise and IL-15 in PC treatment.

### 4.6 IL-21

Although research on IL-21 and pancreatitis is relatively limited, studies have begun to explore the potential connection between IL-21 and PC. It is generally believed that IL-21 exerts anti-tumor effects in PC. Early studies indicated that IL-21 inhibits tumor growth by activating NK cells (166). Recent research has found that in PDAC, the infiltration of T follicular helper (Tfh) cells promotes IL-21 secretion, which helps B cells differentiate into plasma cells, thereby enhancing the humoral immune response (167). Meanwhile, tumor cells inhibit T-cell activation by expressing PD-L1 that binds to PD-1 on T-cells, activating the PD-1/PD-L1 pathway for immune escape (168). However, the PD-L1/PD-1 pathway may inhibit the anti-tumor effects of IL-21. When patients receive neoadjuvant chemotherapy with Nab-paclitaxel and gemcitabine, this inhibition can be reversed, restoring the anti-tumor ability of IL-21 (167). McMichael et al. showed that IL-21 not only significantly enhanced the effector function of NK cells on cetuximab-coated PC cells and increased ADCC, an effect independent of the KRAS gene mutation status of the tumor cells; it also promoted IL-21-activated NK cells to secrete a high level of IFN-γ and chemokines, enhanced T-cell chemotaxis and enhanced NK cell signal transduction by activating the ERK and STAT1 signaling pathways to enhance NK cell signaling; in addition, the combination of IL-21 and cetuximab significantly inhibited the growth of EGFR-positive PCs in a mouse model, and the effect was even better when combined with gemcitabine (169). Giulia Marelli and her team developed a novel virus, VVL21, by embedding IL-21 into a vaccinia virus strain using gene-editing technology (170). This virus enhances the accumulation of effector CD8+T cells within tumors, boosts NK cell activity, and promotes macrophage polarization to the M1 phenotype, offering an effective strategy for clinical PC treatment (170). However, some studies suggest that IL-21 may also play a role in promoting PC invasion. A study by Alica et al. found that high density of IL-21+ immune cells and expression of IL-21R and Blimp-1 in tumor tissues of PDAC patients correlated with poor patient prognosis (171). IL-21 may activate the ERK and STAT3 signaling pathways through binding to IL-21R on the surface of PC cells that which in turn promotes the upregulation of Blimp-1 expression and enhances the invasive ability of tumor cells (171).Therefore, further in-depth research is necessary to understand the complex relationship between IL-21 and PC.

## 5 Discussion

In summary, the IL-2 family plays a complex and multifaceted role in pancreatitis and PC. Unlike IL-1 family members, which act directly as inflammatory mediators, IL-2 family members primarily play a role in immune regulation and inflammatory responses. They may also influence disease progression and response to therapy by regulating the TME and cellular metabolism in the pancreas, which in turn affects disease progression and response to therapy. For example, some IL-2 family members may alleviate pancreatic damage by inhibiting inflammatory responses, while others may aid in restoring pancreatic function by promoting tissue repair. Future research needs to further elucidate the specific mechanisms by which IL-2 family members operate in pancreatic diseases. This includes understanding how they affect the survival, proliferation, and differentiation of pancreatic cells, as well as how they interact with other cell types within the pancreas. Additionally, research should focus on the expression patterns of IL-2 family members at different stages of pancreatic diseases and how they respond to existing treatment methods. As shown in **Tables 1**, **2**.

**Table 1:** Clinical applications of targeted IL-2 family for the treatment of pancreatic diseases.

Cytokines	Existing drug therapies	Mechanisms	Diseases	References
IL-4	Da Cheng Qi Tang	Increased IL-4	AP	(92)
IL-4	Baicalin	Decreased pancreatic alveolar cell pyroptosis	Hyperlipidemic pancreatitis	(93)
IL-4	Thioredoxin 1	Decreased IL-4, M2-type macrophages	CP	(100)
IL-15	rIL-15	Increased NK cells, IFN-γ	CP	(149)
IL-15	IL-15 and metformin	Enhanced tumor cell autophagy and apoptosis	PC	(154)
IL-15	IL-15 and CD40 agonist	Increased CD8+T and NK cells	PDAC	(155)
IL-15	hIL15-ABD and Nab-paclitaxel	Increased CD8+T, NK, and M1-type macrophages; decreased Tregs	PDAC	(157)
IL-9	Mogroside IIE	Decreased histone B and IL-9	AP	(138)
IL-21	Nab-paclitaxel and gemcitabine	Inhibition of PD-L1/PD-1 signaling pathway	PDAC	(167)

**Table 2:** Cellular engineering drugs based on the structure of the IL-2 family.

Cytokines	Existing drug therapies	Mechanisms	Diseases	References
IL-2	PD1-IL2v	Increased CD8+T and Teff cells; Decreased Tregs	PDAC	(85)
IL-4	IL-4/IL-7 ICR	Increased T cells	PC	(111)
IL-4	4/15NKG2D-CAR	Activated IL-15	PC	(112)
IL-4	IL-4/IL-21 ICR	Activated STAT3 pathway	PC	(113)
IL-15	VSV-IL-15	Activated IL-15	PDAC	(158)
IL-15	P-aTIGIT&PDA@IL-15@ge	Increased CD8+T and NK cells	PC	(159)
IL-15	CD70-CAR-IL-15 NK	Decreased fibroblasts	PDAC	(160)
IL-15	PSCA CAR_s15 NK	Increased NK cells	PC	(162)
IL-15	Sushi-IL15-PD1	Enhanced PD-L1 cytotoxicity	PDAC	(163)
IL-9	IL-2Rβ-ECD–IL-9R-ICD	Activated STAT pathway	PC	(146)
IL-21	VVL21	Increased CD8+T and NK cells; Enhanced macrophage M1 polarization	PC	(170)

By conducting in-depth research into the mechanisms of the IL-2 family in pancreatic diseases, scientists hope to discover new biomarkers for early diagnosis and assessment of disease severity. This research could also provide insights for developing new therapeutic strategies, such as modulating the activity of IL-2 family members to regulate immune responses or creating targeted therapies to enhance treatment efficacy and reduce adverse effects. Current research has focused on the immune-activating effects of the family, while relatively few studies have been conducted on its inhibitors. Future studies should also take into account individual differences, such as genetic background and environmental factors, which may influence the role of IL-2 family members. Personalized medicine approaches could adjust the intervention strategies of IL-2 family members based on the specific conditions of patients, aiming for optimal therapeutic outcomes and minimal side effects. In conclusion, research on the IL-2 family in pancreatic diseases is a field filled with challenges and opportunities. As we gain a deeper understanding of the mechanisms of these cytokines, there is hope for developing more precise and effective treatments to improve the prognosis of patients with pancreatitis and PC.
